# A Six-lncRNA Signature for Immunophenotype Prediction of Glioblastoma Multiforme

**DOI:** 10.3389/fgene.2020.604655

**Published:** 2021-01-13

**Authors:** Ming Gao, Xinzhuang Wang, Dayong Han, Enzhou Lu, Jian Zhang, Cheng Zhang, Ligang Wang, Quan Yang, Qiuyi Jiang, Jianing Wu, Xin Chen, Shiguang Zhao

**Affiliations:** ^1^Department of Neurosurgery, The First Affiliated Hospital of Harbin Medical University, Harbin, China; ^2^Key Colleges and Universities Laboratory of Neurosurgery in Heilongjiang Province, Harbin, China; ^3^Institute of Neuroscience, Sino-Russian Medical Research Center, Harbin Medical University, Harbin, China; ^4^Department of General Surgery, The First Affiliated Hospital of Harbin Medical University, Harbin, China; ^5^North Broward Preparatory School, Coconut Creek, FL, United States

**Keywords:** long non-coding RNA, biomarker, immunophenotype, machine learning, glioblastoma multiforme

## Abstract

Glioblastoma multiforme (GBM) is the most aggressive primary tumor of the central nervous system. As biomedicine advances, the researcher has found the development of GBM is closely related to immunity. In this study, we evaluated the GBM tumor immunoreactivity and defined the Immune-High (IH) and Immune-Low (IL) immunophenotypes using transcriptome data from 144 tumors profiled by The Cancer Genome Atlas (TCGA) project based on the single-sample gene set enrichment analysis (ssGSEA) of five immune expression signatures (IFN-γ response, macrophages, lymphocyte infiltration, TGF-β response, and wound healing). Next, we identified six immunophenotype-related long non-coding RNA biomarkers (im-lncRNAs, USP30-AS1, HCP5, PSMB8-AS1, AL133264.2, LINC01684, and LINC01506) by employing a machine learning computational framework combining minimum redundancy maximum relevance algorithm (mRMR) and random forest model. Moreover, the expression level of identified im-lncRNAs was converted into an im-lncScore using the normalized principal component analysis. The im-lncScore showed a promising performance for distinguishing the GBM immunophenotypes with an area under the curve (AUC) of 0.928. Furthermore, the im-lncRNAs were also closely associated with the levels of tumor immune cell infiltration in GBM. In summary, the im-lncRNA signature had important clinical implications for tumor immunophenotyping and guiding immunotherapy in glioblastoma patients in future.

## Introduction

Glioblastoma multiforme (GBM) is the most aggressive type of primary brain tumor in adults, with a median survival of 14.6 months (Kaffes et al., 2019). The emergence of tumor immunotherapy has revolutionized GBM treatment and its success is highly dependent on the development and activation of immune cells in the host microenvironment (Pardoll, 2012; Daud et al., 2016). In the GBM microenvironment, the non-neoplastic cells are mainly from the innate immune system, which can interact with neoplastic tumor cells and play an important role in tumor growth and progression (Engler et al., 2012; Feng et al., 2015; Hu et al., 2015). Therefore, evaluation of GBM tumor immunoreactivity is critical in determining personalized treatment.

Long non-coding RNAs (lncRNAs) are defined as non-coding RNAs of more than 200 nt in length (Zhai et al., 2018). The discovery of lncRNAs has revealed a new dimension to the pathological processes of many diseases, including the occurrence and development of cancer (Martens-Uzunova et al., 2014; Zhou et al., 2014). Moreover, recent studies showed that lncRNAs play an important role in tumor immune escape (Pei et al., 2018; Wang et al., 2019; Jin et al., 2020). For example, UCA1 is able to promote proliferation, migration, immune escape, and inhibit apoptosis in gastric cancer (Wang et al., 2019); SNHG1 can regulate the differentiation of Treg cells and affect the immune escape of breast cancer (Pei et al., 2018). Besides, immune-associated lncRNAs can also serve as improving prognosis and immunotherapy response biomarkers (Zhou et al., 2018; Sun et al., 2020). Therefore, identification of lncRNA biomarkers for tumor immunoreactivity may provide new insights into the treatment of GBM patients.

In this study, we systemically characterized the GBM tumor immune microenvironment in the TCGA GBM cohort. Moreover, we defined the GBM Immune-High (IH) and Immune-Low (IL) subtype based on five immune expression signatures including macrophages, lymphocyte infiltration, TGF-β response, IFN-γ response, wound healing. Furthermore, we identified six immunophenotype-related lncRNA signatures (im-lncRNAs, including USP30-AS1, HCP5, PSMB8-AS1, AL133264.2, LINC01684, and LINC01506) using the minimum redundancy maximum relevance (mRMR) feature selection method and the random forest model. The im-lncRNAs showed good performance in distinguishing tumor immunophenotypes and were closely associated with the levels of tumor immune cell infiltration. These results suggested the im-lncRNAs had the promising potential for clinical diagnosis of GBM immunophenotypes.

## Materials and Methods

### Data Acquisition and Pre-processing

All glioblastoma multiforme tissue samples were obtained from the surgical resection tissue of GBM patients (*n* = 10), non-tumor brain tissue was used as the negative control group (*n* = 5). The tissue samples were stored in liquid nitrogen. All patients have signed informed consent, and the study was supervised and approved by the Ethics Committee of The First Affiliated Hospital of Harbin Medical University.

The Cancer Genome Atlas (TCGA) level 3 gene/lncRNA expression data, and clinical data of GBM (*n* = 149, 144 cancer samples, 5 normal samples) were obtained from the Genomic Data Commons (GDC, available at https://www.cancer.gov/tcga). Two independent datasets GSE79671 (Urup et al., 2017) and GSE121810 (Cloughesy et al., 2019) were used for the validation of im-lncRNAs. For the gene/lncRNA expression data, we removed the genes/lncRNAs that were not expressed over 70% of the samples. The remaining 18,094 genes and 18,567 lncRNAs were used for subsequent analysis.

### Total RNA Extraction and Quantitative Real-Time PCR

According to the manufacturer’s instructions, total RNA was extracted from the GBM tissues and non-tumor brain tissues using TRIzol Reagent (Invitrogen, Carlsbad, CA, United States). The concentration of the total RNA was detected by spectrophotometer (Thermo Scientific^TM^ NanoDrop 2000c). Total RNA (1000 ng) was reverse transcribed into cDNA using qPCR RT Kit (TOYOBO, Japan). The relative level of lncRNAs to the housekeeping gene GAPDH was determined by qRT-PCR using FastStart Universal SYBR Green Master (ROX) (Roche, Germany). All primers used in this study is showed in Supplementary Table 1. Analysis between the two groups was performed by an unpaired *t*-test, *P* < 0.05 was considered statistically significant.

### Identification of Tumor Immune Subtypes of GBM

Based on five immune expression signatures reorganized by Vesteinn et al. (Lek et al., 2016) including IFN-γ response (Wolf et al., 2014), macrophages/monocytes (Beck et al., 2009), overall lymphocyte infiltration (dominated by T and B cells) (Calabro et al., 2009), TGF-β response (Teschendorff et al., 2010), wound healing (Chang et al., 2004), we evaluated the enrichment scores (ESs) of GBM samples using the single-sample gene set enrichment analysis (ssGSEA) (Barbie et al., 2009). The ssGSEA was based on the R package “GSVA.” Furthermore, we used the ESs of immune expression signatures to perform a consensus clustering on 149 cancer samples using the R package “ConsensusClusterPlus” (Monti et al., 2003).

### Evaluation of Tumor Purity, Tumor-Infiltrating Immune Cells, and Cytolytic Activity

The tumor purity of corresponding TCGA samples was evaluated using the ESTIMATE score calculated by the R package “ESTIMATE” (Yoshihara et al., 2013). The higher ESTIMATE score, the lower tumor purity. The tumor immune cell infiltration levels were estimated based on the gene expression profile by Tumor Immune Estimation Resource (TIMER) (Li et al., 2017). Here, six tumor-infiltrating immune cells (B cells, CD4 T cells, CD8 T cells, macrophages, neutrophils, and myeloid dendritic cells) were considered. Cytolytic activity (CYT) was calculated as the geometric mean of the *GZMA* gene and *PRF1* gene (as expressed in FPKM) (Rooney et al., 2015).

### Differential Expression Analysis of lncRNAs

We first calculated the log_2_(fold change) (log_2_(FC)) of each lncRNA between the IL and normal samples, and between the IH and normal samples, respectively. Then we scaled the expression level (log_2_FPKM) of each lncRNA and into a Z-score. Next, we compared lncRNA expression differences between the IL and normal samples, and between the IH and normal samples, using the Student’s *t*-test, respectively. The *P-values* were corrected using the Benjamini-Hochberg adjustment. The lncRNAs with *FDR < 0.01* and *| log_2_FC| > 2* were considered as the differentially expressed lncRNAs.

### Identification of im-lncRNAs

We first divided the GBM cancer samples into three parts (two “training” sets and one “test” set) to apply three-fold cross-validation. Next, we screened the lncRNA features with minimal redundancy using the mRMR feature selection method in the training set (Hanchuan et al., 2005). Further, we trained a random forest model based on the top 5% mRMR lncRNA features. The performance of the random forest model was assessed through prediction making in the test set and the computation of the balanced error rate (BER). For a more robust estimation of the BER, three-fold cross-validation was applied 1,000 times and for each run, randomized data were used as the negative control. The signature size, for which no more improvement of the BER was observed (6 features signature size), was selected as the final size. This process generated 3 × 1000 output signatures. The distance (D) between these signatures was defined as (Jeschke et al., 2017):

(1) $$D=1-\frac{\sum_{i=1}^{6}\mathrm{cor}\left(F1_{i},F2_{i}\right)}{6}$$

where cor represents the Spearman’s correlation coefficient (Rho); F1_*i*_ to the i^*th*^ feature from signature 1 and F2_*i*_ to the i^*th*^ feature from signature 2 after sorting the features to maximize the sum of the Rho based on the changes in the Gini index. For each signature, the sum of its pairwise distance to all other output signatures was computed, and the signature with the smallest sum was assumed to be the most representative and chosen as the final lncRNA signature (im-lncRNA).

### Construction of im-lncScore

To conveniently evaluate the GBM tumor immunophenotypes, we constructed the im-lncScore. Firstly, we applied principal component analysis (PCA) on the Z-scores of im-lncRNAs. Then the first component was used as the final im-lncScore for the cancer samples.

### Analysis of Association Between im-lncRNAs and Tumor Immune Cell Infiltration

Firstly, we calculated the median infiltration levels of each immune cell; if the sample infiltration level was higher than the median level, the sample was defined as a high immune infiltration sample; if the sample infiltration level was lower than the median level, the sample was defined as a low immune infiltration sample. Then, the univariate logistic regression was performed to assess the association between each im-lncRNA and the infiltration levels of each immune cell. The OR, 95% confidence level of the OR, and *P-values* were calculated for each immune cell. The logistic regression was based on the R package “epiDisplay.”

### Identification of Co-expressed Genes With im-lncRNAs

Based on the expression profiles of im-lncRNAs and genes, we calculated the Spearman’s correlation coefficient (Rho) between im-lncRNAs and genes. The raw *P-values* (P_*r*_) were adjusted by multiple hypotheses using a permutation method. For each gene, the expression value (FPKM) was held consistent, and 1,000 random im-lncRNAs were used to perform the same Spearman’s correlation test, generating a set of 1,000 permutation *P-values* (P_*p*_). Finally, an empirical *P-value* (P_*e*_) was corrected using the following formula (which introduces a pseudo-count of 1). The gene with the Rho>0.6 and P_*e*_ <0.01 were treated as the co-expressed genes of im-lncRNAs.

(2) $$P_{e}=\frac{\mathrm{num}\left(P_{p}\leq P_{r}\right)+1}{1001}$$

### Functional Enrichment Analysis

To annotated the biological functions of im-lncRNAs, we performed functional enrichment analysis on the co-expressed genes of im-lncRNAs using Metascape (Zhou et al., 2019). For each co-expressed gene list, pathway and process enrichment analysis have been carried out with the following ontology sources: KEGG Pathway, GO Biological Processes, Reactome Gene Sets, Canonical Pathways, and Hallmark Gene Sets.

## Results

### Characterizing the Immune Microenvironment of GBM

**FIGURE 1:**
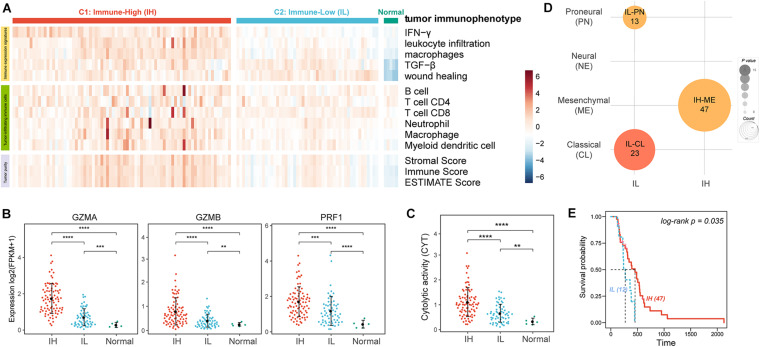
The tumor immune microenvironment of GBM. **(A)** Heatmaps showing the ssGSEA of immune expression signatures (first five lines), the levels of tumor-infiltrating immune cells (sixth to twelfth lines), and tumor purity (last three lines) in GBM patients. **(B)** The expression levels of effectors of immunity log2(FPKM+1). **(C)** The CYT of GBM patients. **(D)** Enrichment analysis of GBM subtypes and GBM immunophenotypes. **(E)** The KM survival curve showing the impact of immune subtypes on the survival of ME GBM patients. **P < 0.01, ***P < 0.001, ****P < 0.0001.

We analyzed 149 GBM RNA-seq expression profiles from TCGA. To evaluate the tumor immune activity, we used a previously described technique employing ssGSEA (Barbie et al., 2009) based on the five immune expression signatures reorganized by Vesteinn et al. (Lek et al., 2016) including IFN-γ response (Wolf et al., 2014), lymphocyte infiltration (Calabro et al., 2009), macrophages/monocytes (Beck et al., 2009), TGF-β response (Teschendorff et al., 2010), wound healing (Chang et al., 2004). The result showed that there were higher ESs of all immune expression signatures in cancer than in normal samples (Figure 1A, IFN-γ *P = 1.01e-03*, leukocyte infiltration *P = 1.83e-04*, macrophages *P = 5.80e-08*, TGF-β *P = 1.55e-05*, and wound healing *P = 3.33e-08*). Moreover, based on the ESs of immune expression signatures, we subclassified the cancer samples using the consensus clustering method. The analysis resulted in 2 robust clusters: C1 and C2. Notably, the ESs of IFN-γ, leukocyte infiltration, macrophages in C1 were significantly higher than in C2 (Figure 1A, IFN-γ *P = 1.46e-29*, leukocyte infiltration *P = 3.43e-18*, and macrophages *P = 2.60e-16*). And, there was no significant difference in the ESs of TGF-β (*P = 3.46e-01*) and wound healing (*P = 1.09e-01*) between the two clusters. Furthermore, we evaluated the levels of tumor purity and tumor-infiltrating immune cells between the two clusters. There were lower tumor purity (*P = 4.75e-16*) and higher percent of tumor-infiltrating immune cells (B cell *P = 1.48e-05*, T cell CD4 *P = 8.26e-4*, Neutrophil *P = 3.58e-06*, and macrophage *P = 7.14e-10*) in C1 than C2 (Figure 1A). Therefore, we annotated the C1 sample was the Immune-High (IH) subtype, and the C2 sample was the Immune-Low (IL) subtype.

To further verify the levels of immune activation in different immune subtypes, we examined the expression levels of common effectors of immunity, such as granzyme A (GZMA), granzyme B (GZMB), and perforin (PRF1) (Figure 1B; Mandal et al., 2016) and the immune cytolytic activity (CYT, an indicator of tumor local immunity, Figure 1C; Rooney et al., 2015). Remarkably, these effectors of immunity and CYT were much higher in the IH subtype compared with the IL subtype.

Glioblastoma multiforme can be subclassified into distinct molecular subtypes based on their expression profiles: classical (CL), mesenchymal (ME), neural (NE), and proneural (PN) (Verhaak et al., 2010; Ceccarelli et al., 2016). Here, we also enriched the tumor immune subtypes into the GBM molecular subtypes using Fisher’s exact test. The previous study indicated ME GBM was the most immunogenic among the four subclasses while the PN subtype was the least immunogenic (Doucette et al., 2013). Our result also showed that ME GBM was significantly enriched in the IH subtype, while CL and PN GBM tumors were significantly enriched in the IL subtype (Figure 1D). Besides, by analyzing the survival of ME GBM patients between IL and IH subtypes, we found the survival of ME with IH patients was significantly better than ME with IL patients (Figure 1E).

### Identification of Immunophenotype-Related lncRNA Biomarkers in GBM

**FIGURE 2:**
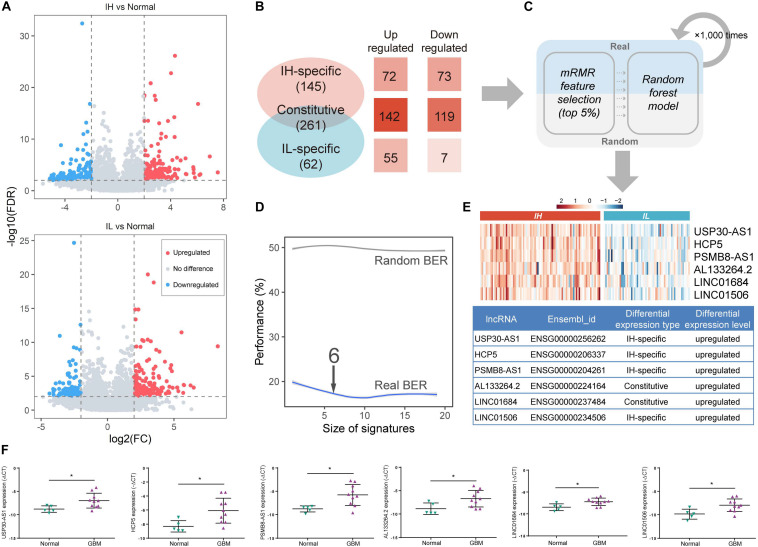
Identification of im-lncRNAs in GBM. **(A)** Volcano plots showing the differentially expressed lncRNAs between IH/IL and normal samples, respectively. The *y*-axis shows the -log_10_(FDR). The *x*-axis shows the log_2_(FC) **(B)** The classification of differentially expressed lncRNAs. **(C)** The pipeline of identifying im-lncRNAs. **(D)** The evaluation of model BER performance. **(E)** Heatmap showing the im-lncRNAs expression in IH and IL samples, respectively. **(F)** The up-regulated six lncRNAs expression in glioblastoma multiforme tissues was confirmed by qRT-PCR (*P < 0.05).

lncRNA, an emerging biomarker, plays an important role in tumor immune regulation (Li et al., 2020). However, few studies focus on the ability of lncRNA in tumor immunophenotyping. To identify the immunophenotype-related lncRNA biomarkers (im-lncRNAs), we first characterized the differentially expressed lncRNAs between the IH/IL and normal samples, respectively (*FDR < 0.01* and *| log_2_FC| > 2*, see section “Materials and Methods,” Figure 2A). We identified 261 “Constitutive” lncRNAs differentially expressed in both immune subtypes (142 upregulated and 119 downregulated), 145 “IH-specific” lncRNAs only differentially expressed in IH subtype (72 upregulated and 73 downregulated), and 70 “IL-specific” lncRNAs only differentially expressed in IL subtype (55 upregulated and 7 downregulated, Figure 2B).

Next, we applied a machine learning method in differentially expressed lncRNAs to identify the im-lncRNAs (Figure 2C). Firstly, under three-fold cross-validation (dividing 144 cancer samples into three parts, two “training” sets [96 samples], and one “test” set [48 samples]), the mRMR feature selection method was used to establish a small signature with minimal redundancy and selected the top 5% lncRNA features to train the random forest models. Next, in the test set, the balanced-error rate (BER) was calculated to evaluate the model performance. For a more robust estimation of the BER, three-fold cross-validation was applied 1,000 times. In each run, randomized data were used as the negative control. The signature size, for which no more improvement of the BER was observed (6 features signature size), was selected as the final size (Figure 2D). This pipeline generated 3 × 1000 output signatures and the signature with the minimum distance summed was assumed to be the most representative (see section “Materials and Methods”). Based on the approach, we identified 6 im-lncRNAs (USP30-AS1, HCP5, PSMB8-AS1, AL133264.2, LINC01684, LINC01506). Notably, USP30-AS1, HCP5, PSMB8-AS1, and LINC01506 were “IH-specific” lncRNAs, and AL133264.2, LINC01684 were “Constitutive” lncRNAs. The expression levels of all im-lncRNAs were significantly higher in IH than IL samples (USP30-AS1 *P = 1.12e-18*, HCP5 *P = 8.07e-15*, PSMB8-AS1 *P = 1.15e-15*, AL133264.2 *P = 4.07e-10*, LINC01684 *P = 1.50e-11*, and LINC01506 *P = 3.00e-10*, Figure 2E). Besides, all of 6 im-lncRNAs were also upregulated in GBM cancer than normal samples, which had been validated by RT-qPCR in five non-tumor brain tissues and ten GBM tissues (Figure 2F). To ensure that the recognized im-lncRNAs were robust, we also employed the same way on an independent dataset [GSE79671 (Urup et al., 2017)]. The results showed that six im-lncRNAs closely associated with the GBM immunophenotypes and four of the six (USP30-AS1, HCP5, AL133264.2, and LINC01506) were consistent with the im-lncRNAs identified in the TCGA GBM cohort (Supplementary Figures 1A–C).

### Evaluation of GBM Tumor Immunophenotyping Efficacy of im-lncRNAs

**FIGURE 3:**
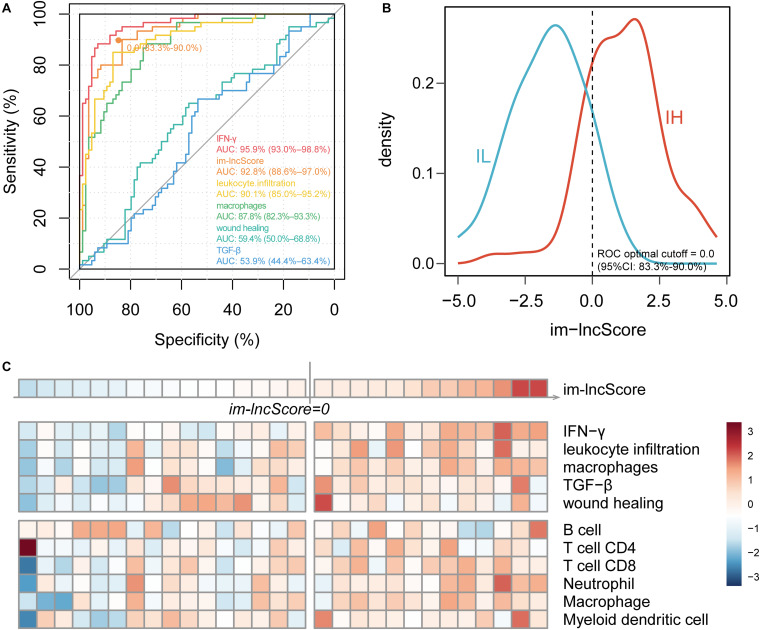
im-lncRNAs enable evaluate GBM immunophenotypes. **(A)** ROC curves for prediction of GBM tumor immunophenotypes based on the im-lncScore (orange) or immune expression signatures (other colors) in the TCGA GBM cohort. **(B)** Density plot showing the distribution of im-lncScore in IH and IL samples. **(C)** Heatmap showing the im-lncScore (the first line), ESs of immune expression signatures (second to sixth lines), and infiltration levels of tumor immune cells (last six lines) in GSE121810 cohort.

To further evaluate the relationship between im-lncRNAs and GBM immunophenotyping, we transformed the individual expression values of the im-lncRNAs into a score (im-lncScore) by applying a principal component analysis (PCA). We assessed the potential of the im-lncScore to predict GBM immunophenotypes in 144 cancer samples. Compared with the immune expression signatures, the im-lncScore also showed a promising performance. An AUC of 0.928 (95% CI, 0.87–0.97) suggested a predictive value for the im-lncScore (Figure 3A). Moreover, the optimal cutoff point determined by the ROC curve analysis was 0.0 (95% CI, 0.83–0.90). We also found that the im-lncScores of IH samples were usually greater than the optimal cutoff, while the opposite was observed for the IL samples (Figure 3B).

Besides, we also validated the immunophenotyping ability of im-lncScore in an independent dataset [GSE121810 (Cloughesy et al., 2019)]. The dataset included 29 GBM samples. We first calculated the im-lncScore to subclassify the GBM samples into IH/IL subtype. 13 IH and 16 IL samples were identified in the dataset (Figure 3C). Next, we also evaluated the ESs of five immune expression signatures and infiltration levels of tumor immune cells. As described above, there were higher ESs of IFN-γ, leukocyte infiltration and macrophages signatures, and higher levels of tumor-infiltrating immune cells in IH than IL samples (Figure 2C). These results suggested that the im-lncScore does not require a complex algorithm to effectively subclassify the GBM tumor immunophenotypes, which also further indicated the important role of im-lncRNAs in GBM tumor immunity.

### Im-lncRNAs Are Associated With the Tumor Immune Cell Infiltration

**FIGURE 4:**
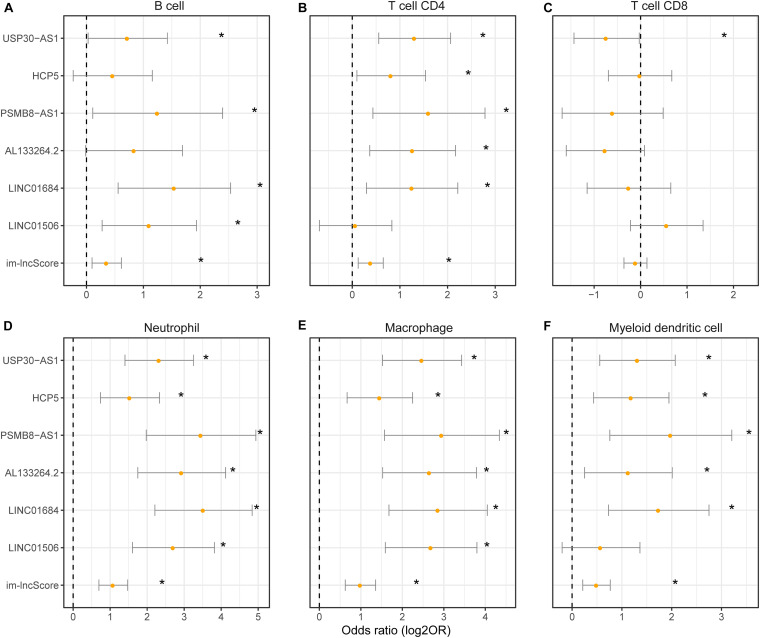
The association between im-lncRNAs and tumor immune cell infiltration. **(A–F)** The im-lncRNAs were correlated with immune cell infiltration. The dots represent the odds ratio (OR) of the Wald test and the error bars show the 95% confidence intervals of the OR. **(A,B)** cells; **(B)** CD4 T cells; **(C)** CD8 T cells; **(D)** neutrophils; **(E)** macrophages; and **(F)** myeloid dendritic cells.

To evaluate whether the im-lncRNAs associated with the levels of tumor immune cell infiltration, we first subclassified the cancer samples into high and low immune infiltration groups by comparing the sample immune infiltration levels to the median immune infiltration level of each immune cell. And then, the univariate logistic regression was performed based on the six im-lncRNAs expression value and im-lncScore. We found that the im-lncRNAs significantly correlated with the infiltration level of multiple immune cells (Figures 4A–F). Notably, the im-lncScore also showed the significantly correlation with multiple immune cell infiltration levels (except for T cell CD8). Besides, HCP5 and PSMB8-AS1 have been demonstrated could be the tumor-infiltrating immune-related lncRNA signature of non-small cell lung cancer and closely associated with outcome and immune cell infiltrates (Sun et al., 2020). These results suggested that the im-lncRNAs played crucial roles in the tumor immune infiltration.

### The Functional Enrichment Analysis of im-lncRNAs

**FIGURE 5:**
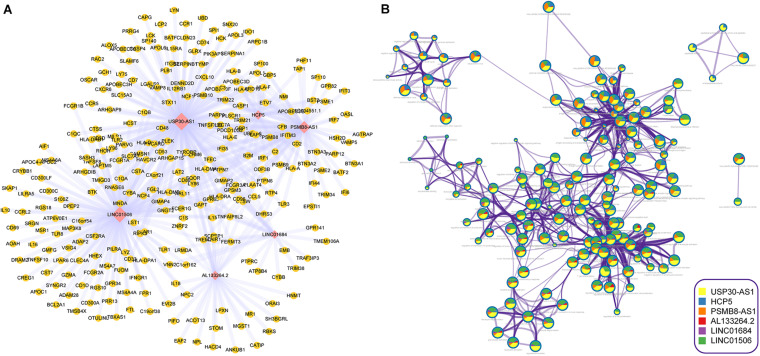
Inferring the biological functions of im-lncRNAs. **(A)** The co-expressed im-lncRNAs-genes network. **(B)** Network of enriched terms represented as pie charts, where pies are color-coded based on the im-lncRNAs.

To further explore the biological functions of im-lncRNAs, we identified the co-expressed genes with the im-lncRNAs using the Spearman’s correlation test. The *P-values* were adjusted by multiple hypotheses. A total of 459 co-expressed im-lncRNA-gene pairs were identified (Figure 5A). Furthermore, we performed functional enrichment analysis on the co-expressed genes using the Metascape (Zhou et al., 2019). The result showed that the functions of co-expressed genes of each im-lncRNAs were all significantly enriched in the immune-related terms, such as hallmark interferon-gamma response (M5913), myeloid leukocyte activation (GO:0002274), tumor necrosis factor superfamily cytokine production (GO:0071706), ER-Phagosome pathway (R-HSA-1236974), etc. (Figure 5B). Moreover, we also found the im-lncRNAs were closely correlated with the GBM-related immune pathways (Li et al., 2020). For instance, the HCP5 and PSMB8-AS1 were related to the Antigen Processing and Presentation, Antimicrobials, and Natural Killer Cell Cytotoxicity; AL133264.2 was related to Interleukins; the LINC01684 and USP30-AS1 were related to Antimicrobials. These results further validated the important role of im-lncRNAs in the GBM immune regulation.

## Discussion

Accumulating evidence suggests that lncRNA serves as a specific molecular marker for tumor immunoreactivity (Wang et al., 2019; Sun et al., 2020). In this study, we analyze the role of lncRNAs in IH and IL tumor immunophenotypes. Moreover, we identify im-lncRNAs based on the machine learning method. Furthermore, we construct an im-lncScore using the expression value of im-lncRNAs. The im-lncScore shows a good performance for distinguishing the GBM tumor immunophenotypes (AUC = 0.928, 95%CI: 0.885–0.970). The im-lncScore does not need a complex algorithm to effectively reflect the patient tumor immunoreactivity. Furthermore, these im-lncRNAs are also closely associated with the levels of tumor immune cell infiltration. This evidence indicates that the im-lncRNAs have the potential to be an important indicator for future clinical diagnosis of GBM immunophenotypes. However, these results are still at the level of the initial calculation, so to ensure accuracy the biology experiments are required to further validate the role of im-lncRNAs. Besides, due to the limited scale, we only use the TCGA data to train our models. Therefore, as the scale of data increases, we will continue to validate the efficiency of im-lncRNAs in GBM.

In summary, im-lncRNAs play an important role in tumor immunophenotyping. Identification of GBM immunophenotypes will provide us a novel insight to improve the therapeutic strategy of GBM. Therefore, the im-lncRNAs has the promising potential for clinical diagnosis of GBM immunophenotypes in the future.

## Data Availability Statement

Publicly available datasets were analyzed in this study. This data can be found here: The Cancer Genome Atlas (TCGA) project (https://www.cancer.gov/tcga), GEO (GSE121810, https://www.ncbi.nlm.nih.gov/geo/query/acc.cgi?acc=GSE121810).

## Author Contributions

MG, XC, and SZ conceived and designed the experiments. MG, XW, DH, and EL analyzed the data. JZ, LW, and QY collected the data. QJ and JW validated the method and data. CZ checked the writing standard of the manuscript. SZ, XC, and MG wrote this manuscript. All authors read and approved the final manuscript.

## Conflict of Interest

The authors declare that the research was conducted in the absence of any commercial or financial relationships that could be construed as a potential conflict of interest.
