# Supplementary material for: A Six-lncRNA Signature for Immunophenotype Prediction of Glioblastoma Multiforme
Source: Front Genet. 2021 Jan 13;11:604655. doi: 10.3389/fgene.2020.604655 (PMC7874158; doi:10.3389/fgene.2020.604655)
Supplement: Supplementary file 1 [file Data_Sheet_1.PDF]

Supplementary Table 1 The primer sequence of qRT-PCR

Term	Primer Sequence (5´-3´)
GAPDH	F:ACCACAGTCCATGCCATCAC
	R:TCCACCACCCTGTTGCTGTA
AL133264.2	F:AGCTGGTGGAACTTGGAGAG
	R:CCCAGCCAGGATCAAGATGA
HCP5	F:CTCCACTCAAAGCTGGCATC
	R:CCTCATGCAAGGAAGGAAGC
LINC01506	F:TCAGTGCTGAGTAGTGGCAAG
	R:AAGCGTCTTGTTCTTCAGCCA
LINC01684	F:TTCCACGGCCTTTAGAACCT
	R:TGCAAGATGTAGCCACCAGA
PSMB8-AS1	F:TGATGGGTCAAGGGTCTTCC
	R:CTGTAACCCATCGCCAAGTG
USP30-AS1	F:GGGATGGGAGAGAGGACTTG
	R:ACCACTCTCCTCGTGATGTC
