# Supplementary material for: A Six-lncRNA Signature for Immunophenotype Prediction of Glioblastoma Multiforme
Source: Front Genet. 2021 Jan 13;11:604655. doi: 10.3389/fgene.2020.604655 (PMC7874158; doi:10.3389/fgene.2020.604655)
Supplement: Supplementary file 2 [file Data_Sheet_2.PDF]

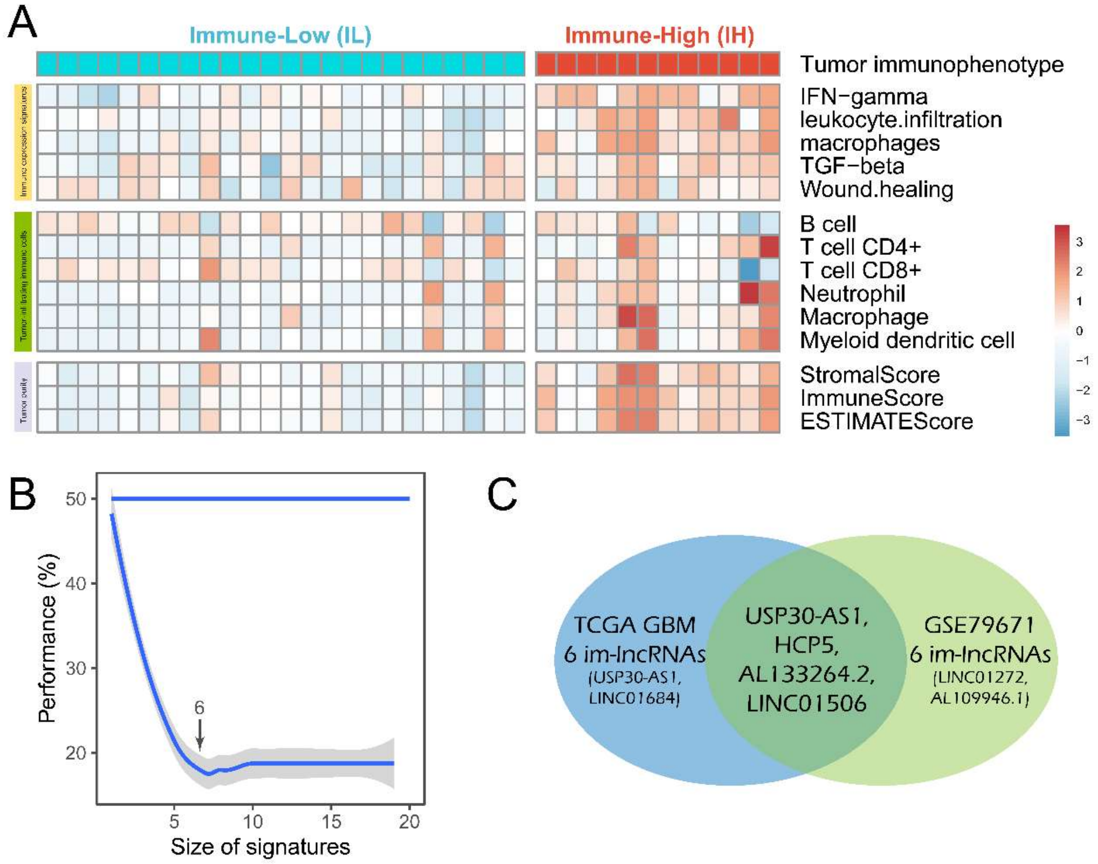

Supplementary Figure 1 The robust validation of im-lncRNAs in GSE79671. (A) The immunophenotypes of tumor samples in GSE79671. (B) The evaluation of model BER performance in GSE79671. (C) Venn diagram showing the number of intersections of im-lncRNAs between the TCGA GBM cohort and the GSE79671.
